# Validation of oral health literacy instrument in Nepali language: a tool to assess oral health literacy of Nepalese population

**DOI:** 10.1186/s12903-026-08632-9

**Published:** 2026-05-19

**Authors:** Tarakant Bhagat, Ashish Shrestha, Santosh Kumari Agrawal, Binita Limbu

**Affiliations:** 1 Department of Public Health Dentistry, College of Dental Surgery, B. P. Koirala Institute of Health Sciences, Dharan, Nepal; 2 Health Directorate, Dhankuta, Koshi Province Nepal

**Keywords:** Oral health literacy, Oral health literacy instrument, Validation study, Nepalese population

## Abstract

**Background:**

Oral health literacy is the capacity of processing and understanding basic oral health information. Instruments are available for its measurement in English and other languages. But no validated tool is available in Nepali language that could be used to assess oral health literacy level of Nepalese population. The objective of this study was to translate and validate the Nepalese version of Oral Health Literacy Instrument (OHLI-N) from the original English version.

**Methods:**

The English version of OHLI along with oral health knowledge test was translated into Nepali language. The translated version was reviewed and pretested on conveniently selected 30 participants. A cross-sectional study was conducted among conveniently selected 160 adult patients of 18 years and above visiting dental OPD of a tertiary care center for testing validity and reliability of OHLI-N. Oral examination was done to record DMFT and CPI scores. Concurrent validity, construct validity and predictive validity of OHLI-N was determined. Internal consistency of OHLI-N was evaluated using Cronbach’s alpha (α). OHLI-N was readministered in 25 patients after 2 weeks to assess test-retest reliability using intra-class correlation coefficient (ICC).

**Results:**

The mean overall OHLI-N score of the participants was 65.9 ± 17.6. OHLI-N scores were significantly associated with educational level of participants (*p* < 0.001). A significant positive correlation (r_s_=0.72) was observed between OHLI-N and oral health knowledge test. Moderate and statistically significant negative correlation was seen between OHLI-N and oral outcomes including DMFT score (r_s_= -0.49), number of untreated caries (r_s_= -0.48), number of teeth with bleeding on probing (r_s_= -0.71) and number of teeth with periodontal pocket (r_s_= -0.49). OHLI-N showed high internal consistency (α = 0.92) and test-retest reliability (ICC = 0.99).

**Conclusion:**

The OHLI-N showed good validity and reliability for assessing the oral health literacy level of adult patients visiting a tertiary care center of Nepal.

## Introduction

Oral diseases, though preventable, are highly prevalent and have remarkable effects on individuals and the society, posing a global public health problem [1]. Oral diseases and oral health inequalities are directly influenced by different social determinants including health literacy [1, 2]. Health literacy has been defined as ‘the degree to which individuals have the capacity to obtain, process, and understand basic health information and services needed to make appropriate health decisions’ [3]. It is a shared function of cultural, social and individual factors that include cognitive abilities, social skills, emotional state, and physical conditions [4]. 

Since the late 1990s, interest in oral health literacy as a domain of health literacy and a factor in health has increased. This interest is driven by the existing oral health disparities with oral diseases affecting particularly the disadvantaged groups [5]. Based on the definition of health literacy, oral health literacy has been defined as the ‘degree to which individuals have the capacity to obtain, process and understand basic oral and craniofacial health information and services needed to make appropriate health decisions’ [6]. Oral health literacy is a key to promoting oral health and preventing oral diseases [5]. People who have poor levels of oral health literacy have poor oral health knowledge, increased emergency dental visits and severity of oral disease [7–12]. Without concurrent improvements in oral health literacy, efforts to increase primary prevention, improve quality of care, reduce costs, and reduce disparities in oral health are bound to fail [13]. 

Assessment of health literacy level is important at the individual and community level. Recognition of low levels of health literacy can help to decide on specific interventions at the policy and private level for improving health outcomes [5]. Currently, there are several instruments that are used to measure health literacy and oral health literacy [14, 15]. But there is no gold standard tool for measuring oral health literacy [15]. The available tools evaluate oral health literacy by two main strategies: word recognition and reading comprehension [15, 16]. Oral health related terms are specific and different from those used in general medicine, thus mandating the development of specific tools to measure an ability to understand dental terms and oral health related information [16]. Initial tools of oral health literacy were adapted from the tools of general health literacy. Most of them are based on either the Rapid Estimate of Adult Literacy in Medicine (REALM) or the Test of Functional Health Literacy in Adults (ToFHLA) [5]. 

Sabbahi et al. [11] developed Oral Health Literacy Instrument (OHLI) for assessment of oral health literacy of adults in 2009 based on the model of ToFHLA. Like ToFHLA, OHLI consists of two sections: reading comprehension and numeracy with 38 and 19 items, respectively. It evaluates functional oral health literacy addressing the ability of reading, understanding and interpreting oral health materials. The instrument was found to have good validity and reliability. OHLI was adapted in Russian [16], Spanish [17] and Malay [18] languages, and it was found to be a valid and reliable oral health literacy assessment tool in those populations.

Most of the oral health literacy tools are developed in English and to date, there is no validated oral health literacy tool in Nepali, which is the native language of Nepal. Adapting English word-recognition instruments to other languages is difficult due to certain linguistic differences [16]. Hence, the Oral Health Literacy Instrument, which uses the reading comprehension method, was chosen, taking in account its good validity and reliability as well. The objective of this study was to translate and validate the original English version of the Oral Health Literacy Instrument into Nepali.

## Materials and methods

### Study setting

It was a cross-sectional study conducted among patients visiting dental OPD of BPKIHS, Dharan. The study was conducted for 1.5 months from 1st October to 15th November 2023. Ethical approval for the study was obtained from the Institutional Review Committee, BPKIHS, Dharan (Ref. No. 158/080/081-IRC).

### Sample size estimation

Sample size was calculated using software nMaster version 2.0 developed by Christian Medical College, Vellore, taking correlation coefficient value of -0.227 between OHLI score and Community Periodontal Index score from the validation study of Cartes-Velasquez and Machuca in Chilean population [17]. Considering the power of this two-tailed study to be 80% and α error of 5%, and adding 10% non-response rate, the final required sample size was 160.

### Study participants

Convenience sampling was done to recruit the study participants. This sampling technique was used to obtain a sample that met our selection criteria with ease. Individuals aged 18 years and above, who could read, speak, and understand Nepali language properly, were included in the study. The exclusion criteria were those with intellectual and/or physical disabilities who could not fill the pro forma and those who were not willing to participate in the study.

### Data collection tools

For data collection, a pro forma was used that comprised four sections, including:

1. Sociodemographic characteristics and frequency of dental visit.
2. Oral health knowledge test.
3. Oral health literacy instrument (OHLI-N) with its reading comprehension and numeracy sections.
4. Recording form for DMFT and CPI.

### Oral health literacy instrument

The original English version of Oral Health Literacy Instrument (OHLI) given by Sabbahi et al. [11] was translated in Nepali version. It consisted of two sections: (a) reading comprehension section and (b) numeracy section comprising of 38 items and 19 items, respectively with total of 57 items. The reading comprehension section had two passages with omitted words, one on dental caries and the other on periodontal disease. Four possible choices were given, one of which was correct. The numeracy section included a series of printed prompts: five labels with directions for taking common prescriptions associated with dental treatment, a post-extraction instruction and a dental appointment card. There were 19 questions in total related to the prompts.

### Oral health knowledge test

It evaluated patient’s general dental knowledge and consisted of seven pictures depicting 17 labelled items such as perioral and intraoral structures, oral diseases and conditions, dental fillings, dental prosthesis and different oral hygiene aids. There was a list of numbered words to the left of each picture and participants were asked to match the labels on picture with the numbered item.

### Translation

The English version of OHLI was translated into Nepali language and validity was checked by the back translation method following the guidelines by Beaton et al. [19] The process started with forward translation of the instrument from English into Nepali language by two translators independently. The first translator was informed about the concepts assessed by the OHLI before translation. The second translator was a naive translator with no dental background who was unaware of the concepts. The two translations were evaluated by a review committee comprising the investigators of the study, and combined to form a single Nepali version (OHLI-N). The translated version was then sent to two different translators who were fluent in both English and Nepali for back translation to English language. Both of them did not have dental background and were not informed about the concepts assessed by the OHLI. The review committee assessed the equivalence between the English and Nepali versions. The oral health knowledge test was translated and adapted following the same process. Permission was obtained from Dr. Sabbahi to translate the OHLI into Nepali language. The OHLI-N is available upon request from the corresponding author.

The OHLI-N and oral health knowledge test were pretested on a convenience sample of 30 patients to detect any misunderstandings, ambiguities or other difficulties that the patients could encounter with the instrument items. Minor modifications were made. Short explanation of dental caries, composite and amalgam was added to make them clearer. The word used for attachment loss seemed to be confusing and hence was modified to make it understandable to the intended population.

### Data collection

Patients visiting the dental OPD of BPKIHS, Dharan were approached and recruited conveniently from the waiting room after assessing for eligibility. Written informed consent was obtained from eligible patients who were willing to participate in the study. They were informed about the purpose of the study and also instructed on how to fill the oral health knowledge test and comprehension section of OHLI-N. A pro forma was used to collect information on their sociodemographic characteristics including age, gender, level of education and frequency of dental visits.

The comprehension section of oral health literacy instrument and oral health knowledge test were self-administered to the participants. For numeracy section of OHLI-N, they were shown the prompts and asked related questions which they had to answer orally.

### Score calculation

Each correct answer received a mark (1), and incorrect or no answer received zero mark (0) for both the OHLI-N and the oral health knowledge test. The raw scores of the reading comprehension and numeracy sections were then multiplied by 1.316 (50/38) and 2.632 (50/19), respectively, to create a weighted score ranging from 0 to 50 for each section. The total score for the OHLI-N ranged from 0 to 100. Similarly, the oral health knowledge raw score was multiplied by (100/17) 5.88 to create a weighted score ranging from 0 to 100. The levels for OHLI-N scores and oral health knowledge test scores were established as:

- 0–59: inadequate
- 60–74: marginal
- 75–100: adequate

### Oral examination

After the administration of the instrument, oral examination was done by single examiner (BL) as per WHO guidelines to record DMFT index and Community Periodontal Index (CPI) using a mouth mirror and CPI probe under artificial LED light [20]. DMFT score, number of untreated caries, number of teeth with bleeding on probing, and number of teeth with periodontal pocket ≥4 mm were recorded.

### Validity and reliability of OHLI-N

#### Validity

Face validity and content validity of OHLI-N were assessed during the translation process. Construct validity was assessed by correlating the scores of OHLI-N with the scores of oral health knowledge test. The patients with low oral health knowledge score would be more likely to have low oral health literacy score. Pearson’s/Spearman correlation was used to measure the construct validity. Concurrent validity was measured by comparing OHLI-N scores across categories of education level and frequency of dental visits. People with low oral health literacy would be likely to have lower level of education and make a dental visit less frequently or only when they suffer from pain. Predictive validity was assessed by determining the correlation between OHLI-N scores and oral health outcomes assuming that low oral health literacy is associated with poor oral health. Oral health outcomes included DMFT score, number of untreated caries, number of teeth with bleeding on probing and number of teeth with periodontal pocket ≥4 mm.

#### Reliability

Internal consistency of the OHLI-N and the oral health knowledge test was assessed by calculating Cronbach’s alpha. Twenty-five patients having second appointment for their treatment were retested after two weeks to check test-retest reliability of OHLI-N and oral health knowledge test as well as the intra-rater reliability of the investigator for the oral examination. Intra-class correlation coefficient (ICC) values were calculated for OHLI-N, oral health knowledge test, DMFT, and CPI scores.

### Statistical analysis

The data collected were entered in Microsoft excel and then imported to Statistical Package for Social Sciences (SPSS) version 20.0 for analysis. Differences in mean scores of OHLI-N among different categories of gender, educational level and frequency of dental visit were tested using Independent t-test and ANOVA as the data were found to be normally distributed using Shapiro-Wilk test. The data of the variables- oral health knowledge test score, DMFT score, number of untreated caries, number of teeth with bleeding on probing and number of teeth with periodontal pocket ≥4 mm did not show normal distribution. So, the correlation of OHLI-N with these variables was assessed by calculating Spearman’s correlation coefficient. The level of significance was set at *p* < 0.05.

Internal consistency of the Nepalese version of OHLI instrument and the oral health knowledge test was assessed by calculating Cronbach’s alpha. Test-retest reliability was evaluated using the intra-class correlation coefficient (ICC).

## Results

**Fig. 1:**
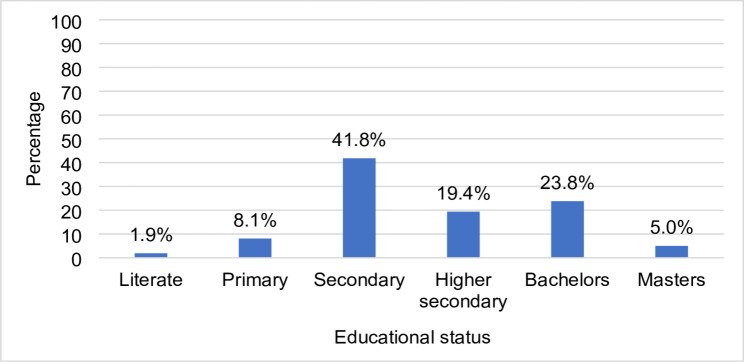
Distribution of participants according to level of education

**Fig. 2:**
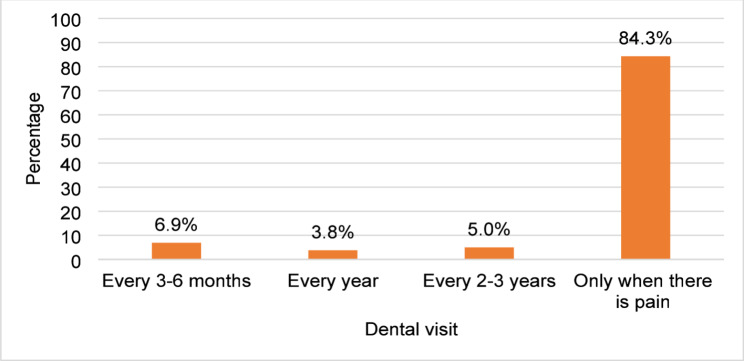
Distribution of study participants according to their frequency of dental visit

A total of 160 participants were included in this study for the validation of OHLI-N. The age of the participants ranged from 18 to 70 years with mean of 34.1 ± 12.8 years. Majority of the participants were male (63.1%). Nearly 42% of the participants had completed secondary level of education whereas about 2% of them had not done any schooling but could read and write in Nepali language (Fig. 1). Highest proportion of the participants (84.4%) visited a dentist only when they had dental pain (Fig. 2).

The mean oral health knowledge test score of the participants was 59.8 ± 19.1. Half of them had inadequate oral health knowledge (oral health knowledge test score < 60). The score was 56.30 ± 20.06 for male and 65.86 ± 15.79 for female participants. The overall mean OHLI-N score was 65.9 ± 17.6 which indicated that they had marginal oral health literacy. One-third of the participants had inadequate (OHLI-N score < 60) and one-third had adequate (OHLI-N score ≥ 75) oral health literacy. In oral assessment, mean DMFT score and mean number of untreated caries were found to be 3.6 ± 2.8 and 2.3 ± 1.8, respectively. Likewise, mean number of teeth with bleeding on probing was 17.9 ± 8.3 and periodontal pocket was 1.8 ± 3.3. The descriptive statistics related to oral health knowledge test, OHLI-N and oral health variables are presented in Table 1.

**Table 1:** Descriptive statistics of oral health knowledge test, oral health literacy instrument and oral health variables (*n* = 160)

Variables	Mean (SD)
Oral health knowledge score	59.8 (19.1)
Reading comprehension score	31.1 (8.6)
Numeracy score	34.7 (10.1)
OHLI-N score	65.9 (17.6)
DMFT score	3.6 (2.8)
Number of untreated caries	2.3 (1.8)
Number of teeth with bleeding on probing	17.9 (8.3)
Number of teeth with periodontal pocket	1.8 (3.3)
Oral health knowledge level	[n (%)]
Inadequate	82 (51.2)
Marginal	41 (25.6)
Adequate	37 (23.1)
Oral health literacy level	[n (%)]
Inadequate	53 (33.1)
Marginal	52 (32.5)
Adequate	55 (34.4)

*SD* Standard deviation

### Validation of Nepali version of OHLI

OHLI-N scores were compared across categories of gender, educational level and frequency of dental visit. Both male and female participants had comparable oral health literacy score. There was an increase in OHLI-N score with increase in the educational level and it was statistically significant (*p* < 0.001). This showed that level of education and oral health literacy are associated thus providing evidence of concurrent validity. When compared with the frequency of dental visit, OHLI-N score was found to be lowest in those who visited dentist only when there was pain. Those visiting dentist every 3–6 months had highest OHLI-N score. However, the difference in OHLI-N scores in different categories of frequency of dental visit was not statistically significant (*p* = 0.102) (Table 2).

**Table 2:** Comparison of OHLI-N scores by gender, education level and frequency of dental visit (*n* = 160)

Variable	*N*	OHLI-*N* score [Mean (SD)]	*P*-value
Gender			
Male	101	64.2 (18.5)	0.11^α^
Female	59	68.8 (15.9)	
Level of education			
Literate	3	41.7 (5.03)	**< 0.001** ^ꞵ^
Primary	13	44.5 (16.5)	
Secondary	67	63.1 (16.1)	
Higher secondary	31	65.1 (14.6)	
Bachelors	38	77.6 (13.6)	
Masters	8	80.3 (9.9)	
Frequency of dental visit			
Every 3–6 months	11	77.4 (16.9)	0.10^ꞵ^
Every year	6	69.8 (21.8)	
Every 2–3 years	8	70.1 (18.1)	
When there is pain only	135	64.5 (17.3)	

Bold indicates statistically significant values

*SD* Standard deviation

^α^unpaired t-test, ^ꞵ^ANOVA test

Construct validity of OHLI-N was assessed by correlating it with oral health knowledge test score. A positive correlation (r_s_=0.72) was observed between them, which was statistically significant. For predictive validity, OHLI-N score was correlated with scores of DMFT and CPI components. Moderate and statistically significant negative correlation was seen between OHLI-N and oral health variables including DMFT score (r_s_= -0.49), number of untreated caries (r_s_= -0.48), number of teeth with bleeding on probing (r_s_= -0.71) and number of teeth with periodontal pocket (r_s_= -0.49). It showed that people with higher oral health literacy level had better oral health (Table 3).

**Table 3:** Construct validity and predictive validity of OHLI-N

	Spearman’s correlation coefficient (*r*_s_)	CI (lower limit, upper limit)	*P*-value
OHLI-*N* and oral health knowledge test	0.72	0.63, 0.79	**< 0.001**
OHLI-N and DMFT	-0.49	-0.62, -0.35	**< 0.001**
OHLI-N and number of untreated caries	-0.48	-0.62, -0.33	**< 0.001**
OHLI-N and number of teeth with bleeding on probing	-0.71	-0.79, -0.61	**< 0.001**
OHLI-N and number of teeth with pocket	-0.49	-0.61, -0.37	**< 0.001**

Bold indicates statistically significant values

*CI *Confidence Interval

Cronbach’s alpha coefficient (α) of 0.72 and 0.92 was obtained for oral health knowledge test and OHLI-N, respectively indicating high degree of internal consistency and homogeneity of both instruments. Intra-class correlation coefficient (ICC) values calculated for oral health knowledge test and oral health literacy instrument among 25 participants reassessed after 2 weeks were also high (> 0.9) showing excellent test-retest reliability (Table 4). Intra-rater reliability of the examiner was assessed for oral examination. The ICC values for DMFT, number of untreated caries, number of teeth with bleeding on probing and number of teeth with periodontal pocket calculated among the same participants were 0.99, 0.94, 0.97 and 0.98, respectively. This showed that the measurements made by the examiner were reliable.

**Table 4:** Internal consistency and test-retest reliability of oral health knowledge test and OHLI-N

	Number of items	Cronbach’s alpha (α) (*n* = 160)	Test-retest correlations (*n* = 25)
Oral health knowledge test	17	0.72	0.97
Reading comprehension	38	0.85	0.98
Numeracy	19	0.87	0.99
OHLI-N	57	0.92	0.99

## Discussion

Oral health literacy is the capacity to access, comprehend, and apply oral health information in appropriate manner [21]. People who have improved oral health literacy are supposed to be better in managing and taking care of their own oral health [22]. Assessing oral health literacy level and linking it with oral health requires a reliable measurement tool. This study was undertaken to translate and validate the Nepali version of Oral Health Literacy Instrument (OHLI-N) that can be used for measuring the functional oral health literacy of Nepali population in the future.

The commonly used oral health literacy instruments are REALD-30 (Rapid Estimate of Adult Literacy in Dentistry) and TOFHLiD (Test of Functional Health Literacy in Dentistry). REALD-30 is only a word recognition test that does not assess reading comprehension ability. Though it is simple and quick tool that can be used in clinical setting, it is inadequate for oral health literacy assessment for research and intervention purposes. This tool also has low predictive validity [23]. TOFHLiD is another instrument that assesses functional oral health literacy and has comprehension test as well [24]. But some of the contents of this instrument related to Medicaid rights are not applicable in case of Nepal. In addition, TOFHLiD is targeted to parents of pediatric dental patients with oral health context specific to pediatric dentistry. None of the oral health literacy tools have been translated into Nepali language. OHLI was chosen for translation and validation in Nepali language since it uses both comprehension and numeracy tests to assess functional oral health literacy. It estimates patient’s ability to carry out oral health literacy-related tasks that need reading comprehension and numeracy skills. Unlike TOFHLiD, OHLI can be applied to the general adult patient population since its content is related to general oral health [11]. 

Translating OHLI tool in Nepali language presented cultural and contextual challenges. English language is more direct and explicit compared to Nepali language, hence sentences in the OHLI tool were translated making them more culture-appropriate. Certain terms such as composite, frenum, dental floss, plaque, clinical attachment, do not have direct Nepali equivalents and are not commonly used in Nepali culture. So, we added short explanations, wherever possible, to make them understandable. The “refill” part in prescription prompts of numeracy section was replaced with a sentence stating ‘no need to further add the medicine’ in Nepali.

In our study, the translated Nepali OHLI showed good validity and reliability. Those with lower level of education had lower oral health literacy scores as expected and the difference was statistically significant, illustrating concurrent validity of OHLI-N. This result was similar to that of original OHLI validation study [11] and of the Russian [16] and Malay [18] versions of the instrument. Such association between educational level and oral health literacy have been seen in many other studies that used different tools to assess the oral health literacy level [9, 25, 26]. Educational level and oral health literacy are influenced by similar socioeconomic and cultural concepts and the level of education can be a social determinant directly affecting the level of oral health literacy [27]. Individuals with lower oral health literacy are more likely to visit dentist less frequently [26, 28]. In this study, those who made a dental visit in case of pain only had lower level of oral health literacy compared to those who saw dentist at regular intervals. However, the difference in OHLI-N scores in different categories of dental visit frequency was not significant. This was contrary to the results of the original English version, Russian and Malay versions of OHLI. The reason for this could be the smaller number of participants visiting dentist in regular intervals in our study resulting in failure to detect significant differences between the categories. In addition, even those individuals with high oral health literacy might have visited dentist only when in pain due to financial barrier. This could be the reason for the obtained result.

Oral health knowledge test used in the original OHLI validation study was used in our study. Health knowledge is regarded either as a domain or a predisposing factor for health literacy [29]. For evaluation of construct validity of OHLI-N, we had assumed positive association between oral health knowledge and oral health literacy based on Baker’s model. This model of health literacy views conceptual knowledge as a person’s resource that facilitates health literacy but not as a constituent of health literacy itself [29]. Limited oral health literacy has been reported to be significantly associated with lower oral health knowledge [30]. Positive and significant correlation obtained between these two parameters in this study, which was stronger than that of the original version [11] (r_s_=0.573), Chilean version [17] (r_s_=0.690) and the Russian version [16] (r_s_=0.363) showed that OHLI-N had good construct validity. Since Nepali language is more comprehensive, stronger correlation might have been obtained in Nepali compared to the English version.

Oral health literacy is considered as a predictor of oral health. Limited oral health literacy is positively associated with poorer oral health outcomes [30]. Individuals with low oral health literacy are at increased risk to oral diseases and the problems related to those diseases [9]. Oral health literacy influences the oral health related decisions of individuals, that in turn determines their overall oral health [31]. Thus, predictive validity of OHLI-N was assessed in our study using oral health outcomes. Significant negative correlation was observed between OHLI-N score and all the oral health variables. Those with lower oral health literacy were likely to have higher number of decayed teeth, teeth with bleeding on probing and teeth with periodontal pocket. This showed that poor oral health literacy is associated with poor oral health. Similar result was observed in the Chilean validation study as OHLI scores were found to be negatively correlated with DMFT and CPI scores [17]. However, in the Malay OHLI, the literacy scores were not found to be correlated with any of the clinical variables [18]. 

The Nepalese version of OHLI showed high internal consistency and test-retest reliability like the original English OHLI. For internal consistency, the value of Cronbach’s alpha was higher than that observed in the English [11] (0.854), Russian [16] (0.895), Chilean [17] (0.887) and Malay [18] (0.88) versions. Similarly, the intra-class correlation coefficient value for OHLI-N was also higher in this study as compared to the original version and other translated versions showing high temporal stability of the instrument in measuring oral health literacy among Nepali population visiting a tertiary care center.

## Strengths and limitations

This is the first study undertaken to translate and validate an oral health literacy assessment tool in Nepali language. This tool can be used for measuring the oral health literacy level of Nepalese adult population. One limitation of the original study was not evaluating the predictive validity of OHLI which was done in this study for OHLI-N. This further adds to the strength of validation of the instrument.

This study has several limitations. We used a convenience sample for our study, selected from patients visiting one dental hospital. This can limit the generalizability of the instrument among the general population, especially for those who have never visited a dentist. Predictive validity was assessed in terms of clinical outcomes only and not with respect to oral health-related quality of life. It takes about 20–30 min to administer OHLI-N. This could limit its use in clinical settings and field surveys, where target population do not have much time to spare. OHLI-N is more appropriate as a research tool.

OHLI-N tool, similar to the original OHLI, focuses on reading and numeracy while other dimensions of oral health literacy such as communication skills and decision-making ability may not be fully reflected. This instrument can only be used in those individuals who can read and write in Nepali language. Those who cannot do both are excluded thus limiting the use of the tool in low-literate population.

## Conclusion

The OHLI-N has good validity and reliability when used among adult dental patients. Future research can be conducted in other parts of the country and in communities to further add to the evidence of validity of the instrument among general Nepali population. Since Nepal is a country with ethnolinguistic diversity, OHLI-N tool can be tested in different communities and ethnic groups and modifications can be made. Considering the results of this study, OHLI-N can be used to assess the oral health literacy level of adult population of Nepal. This can be an important step to further assess various factors that can influence their oral health literacy and plan on intervention programs for its improvement.

## Data Availability

The data collected and analysed in this study are available on request from the corresponding author.

## Abbreviations

ANOVA: Analysis of Variance
BPKIHS: B.P. Koirala Institute of Health Sciences
CPI: Community Periodontal Index
DMFT: Decayed Missing Filled Teeth
ICC: Intraclass Correlation Coefficient
OHLI: Oral Health Literacy Instrument
OHLI-N: Nepali Oral Health Literacy Instrument
REALD-30: Rapid Estimate of Adult Literacy in Dentistry-30
SD: Standard Deviation
TOFHLiD: Test of Functional Health Literacy in Dentistry
